# Contribution of Viral Genomic Diversity to Oyster Susceptibility in the Pacific Oyster Mortality Syndrome

**DOI:** 10.3389/fmicb.2020.01579

**Published:** 2020-07-10

**Authors:** Jean Delmotte, Cristian Chaparro, Richard Galinier, Julien de Lorgeril, Bruno Petton, Pierre-Louis Stenger, Jeremie Vidal-Dupiol, Delphine Destoumieux-Garzon, Yannick Gueguen, Caroline Montagnani, Jean-Michel Escoubas, Guillaume Mitta

**Affiliations:** ^1^IHPE, Université de Montpellier, CNRS, Ifremer, Université de Perpignan Via Domitia, Montpellier, France; ^2^LEMAR UMR 6539, Université de Bretagne Occidentale, CNRS, IRD, Ifremer, Argenton-en-Landunvez, France

**Keywords:** *Crassostrea gigas*, herpesvirus diversity, genotype-genotype interactions, oyster genetic background, viral populations

## Abstract

Juvenile Pacific oysters (*Crassostrea gigas*) are subjected to recurrent episodes of mass mortalities that constitute a threat for the oyster industry. This mortality syndrome named “Pacific Oyster Mortality Syndrome” (POMS) is a polymicrobial disease whose pathogenesis is initiated by a primary infection by a variant of an Ostreid herpes virus named OsHV-1 μVar. The characterization of the OsHV-1 genome during different disease outbreaks occurring in different geographic areas has revealed the existence of a genomic diversity for OsHV-1 μVar. However, the biological significance of this diversity is still poorly understood. To go further in understanding the consequences of OsHV-1 diversity on POMS, we challenged five biparental families of oysters to two different infectious environments on the French coasts (Atlantic and Mediterranean). We observed that the susceptibility to POMS can be different among families within the same environment but also for the same family between the two environments. Viral diversity analysis revealed that Atlantic and Mediterranean POMS are caused by two distinct viral populations. Moreover, we observed that different oyster families are infected by distinct viral populations within a same infectious environment. Altogether these results suggest that the co-evolutionary processes at play between OsHV-1 μVar and oyster populations have selected a viral diversity that could facilitate the infection process and the transmission in oyster populations. These new data must be taken into account in the development of novel selective breeding programs better adapted to the oyster culture environment.

## Introduction

Bivalve mollusk culture is an important sector of world aquaculture, representing approximately a fifth of the global production with 15.7 million tons harvested in 2017 (FAO and WHO, 2018). Given the economic scale and growing importance as a food source for the human population of bivalve farming, much interest has been devoted to investigate infectious diseases, representing a main limitation for aquaculture expansion (Stentiford et al., 2012; Pernet et al., 2016; Burge et al., 2018). Among pathogens, viruses represent major risks for the sustainable management of this sector, as some viruses are highly infectious, easily transmissible, and bear a high extinction potential (Guo and Ford, 2016). In particular, in the 1970s, an infection with an iridovirus completely decimated the population of *Crassostrea angulata* oysters, then cultivated on all European coasts (Comps et al., 1976; Renault and Novoa, 2004; Arzul et al., 2017). Currently, the Ostreid-1 herpesvirus (OsHV-1) is recognized as being responsible for sporadic mortalities in several bivalve mollusks but it is one of the main mortality threats for the most cultivated bivalve species, the Pacific oyster *Crassostrea gigas* (Arzul et al., 2001a; Renault et al., 2001; Davison et al., 2005; da Silva et al., 2008; Solomieu et al., 2015; Xia et al., 2015; Burge et al., 2018). In this bivalve species, OsHV-1 triggers the Pacific oyster mortality syndrome (POMS) that has plagued the oyster production worldwide, from Europe to north and south America and Asia, for more than a decade (Pernet et al., 2012; Hwang et al., 2013; Jenkins et al., 2013; Bai et al., 2015; Mortensen et al., 2016; Prado-Alvarez et al., 2016; Caceres-Martinez et al., 2018). This syndrome, affecting juveniles, leads to mass mortalities that can reach 100% within days (Segarra et al., 2010). Research efforts have revealed a series of factors contributing to the disease, including infectious agents interacting with seawater temperature and oyster genetics (Segarra et al., 2010; Pernet et al., 2012; Petton et al., 2013; EFSA, 2015; Petton et al., 2015; Le Roux et al., 2016; Azema et al., 2017). Recently, holistic molecular approaches uncovered the pathogenis associated to POMS (de Lorgeril et al., 2018). These studies showed that an infection by a variant of OsHV-1 (Oyster herpesvirus type 1 variant μVar) is the critical step of the infectious process leading to an immune-compromised state followed by a microbiota destabilization that “opens the door” to bacterial pathogens (e.g., vibrios) that target haemocytes to induce their lysis (Rubio et al., 2019). The infectious process is completed with subsequent bacteraemia ultimately leading to oyster death (de Lorgeril et al., 2018).

Although the first description of herpes-like viral infection in oyster (*Crassostrea virginica*) was reported in 1972 by Farley and collaborators (Farley et al., 1972), it was not until the 2000s that partial genome characterization allowed the identification of Ostreid herpesvirus 1 (OsHV-1), a new viral species infecting Pacific oyster larvae and spats (Le Deuff et al., 1994). The first variants of the virus identified, called OsHV-1 var, were associated with mortalities in *C. gigas* and other mollusks; they notably carried a deletion of 2.8 kpb as compared to the previous, not fully assembled, OsHV-1 genome (Arzul et al., 2001c). The genome of OsHV-1 has been fully sequenced from oyster larvae in 2005 and contains a double strand DNA genome of ∼207 kbp encoding approximately 124 open reading frames (ORFs) (Davison et al., 2005). Then in 2008, an emergent genotype, called OsHV-1 μVar, was characterized. It was associated with higher mortalities (Segarra et al., 2010) and several variants of μVar genotype have been identified over the last decades (reviewed in Solomieu et al., 2015).

The OsHV-1 μVar genotype has a 12 bp deletion in a microsatellite locus upstream of ORF4, indel/substitution in ORFs 4/42/43, and a complete loss of ORFs 36/37 as well as partial loss of ORF 38 (Segarra et al., 2010; Renault et al., 2012; Martenot, 2013). Over the past 5 years, with the increasing use of sequencing technologies, more and more genotypes were identified such as OsHV-1-SB (Xia et al., 2015) OsHV-1 μVar A and B genotypes with insertion of 4 ORFs including one coding a membrane protein (Burioli et al., 2017) OsHV-1-PT from the North Adriatic Sea (Abbadi et al., 2018) and OsHV-1 isolate ZK0118 (Bai et al., 2019). In addition, PCR amplification of a subset of ORFs allowed the screening of new genotypes (Burioli et al., 2018). This expanding number of OsHV-1 genotypes and number of carrier species (Arzul et al., 2001a, b, 2002; Batista et al., 2007; Bai et al., 2015) raises the question of the impact of this genetic diversity on spreading and severity of POMS.

Accumulating research over the past decades have shown that a large number of viruses exhibit a high level of genetic diversity. This is especially true for viruses with RNA genomes, including medically important viruses such as HIV, hepatitis C virus, and influenza; this is largely due to the low fidelity of their viral RNA-dependent RNA polymerase (RdRp) (Drake and Holland, 1999; Parvez and Parveen, 2017). Extensive study of this diversity has led to the development of the quasispecies concept that questioned our current understandings of viral diseases and their evolution (Batschelet et al., 1976; Vignuzzi et al., 2006; Lauring and Andino, 2010). It seems that many viruses have the ability to produce diverse genetically linked mutants that can be defined as viral populations or quasispecies maintained by mutation-selection equilibrium (Perales et al., 2015; Poirier and Vignuzzi, 2017). This concept hypothesizes that dynamic viral populations generate beneficial interactions and cooperation that allows them to rapidly adapt to changing environments, including various hosts and their different tissues, relevant for viral fitness and virulence (Xiao et al., 2016; Brooke, 2017). Studies have shown that the level of genetic diversity within viral populations likely influences viral pathogenicity, dissemination and host immune evasion (Mao et al., 2007). Conversely, limited diversity could strongly attenuate virulence (Pfeiffer and Kirkegaard, 2005). DNA viruses also bear genomic variability that rivals that of many RNA viruses (Renzette et al., 2011; Renner and Szpara, 2018). Recent work has shown a significant inter-host and intra-host genetic divergence across tissue compartments and time of infection by herpesviruses (Renzette et al., 2013, 2014). In addition, the evolution of a disease is sometimes not fully explained by intrinsic factors of the host, but rather by the variability of herpesvirus (Akhtar et al., 2019).

Up to now, far too little attention has been paid to the role of OsHV-1 genetic diversity in POMS. Here, we analyzed a set of biparental oyster families displaying contrasting susceptibility phenotypes to POMS in two different infectious environments. This biological material gave us the unique opportunity to explore the impact of the genetic diversity of OsHV-1 μVars on the outcomes of the disease for different oyster genotypic backgrounds. We found that susceptibility phenotypes of oyster families varied from one infectious environment to another. Single Nucleotide Polymorphism (SNP) exploration from RNAseq data revealed different viral populations in the different infectious environments; these viral populations were different from the reference OsHV-1 μVar genome. Moreover, in a same infectious environment, the different oyster families were shown to be infected by different OsHV-1 variants. The genetic diversity of viruses between oyster families and environments suggests a host-pathogen co-evolutionary process. Interestingly, viral populations contain protein-coding variations that might impact viral pathogenesis, as revealed by SNPs localization. This work advances our knowledge of OsHV-1 μVar diversity and constitutes a step toward future investigation on the role of viral genetic variation, its relationship with phenotype, host genetics, and impact on disease outcome.

## Materials and Methods

### Production of Biparental Oyster Families

**FIGURE 1:**
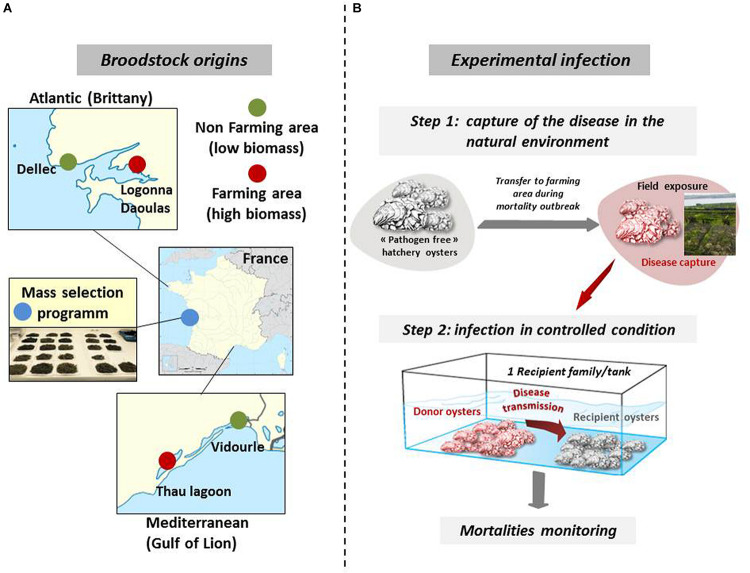
Broodstock origins for the production of biparental oyster families and schematic of experimental infection. **(A)** Wild stocks were sampled in farming (red) and non-farming (green) sites in two geographic areas (Atlantic and Mediterranean coasts). Mass selected oysters (blue) originated from the Ifremer hatchery of La Tremblade. Image source: commons.wikimedia.org. **(B)** For experimental infection, pathogen free oysters were deployed in the natural environment in a farming area during disease outbreaks and brought back to a controlled environment to transfer the disease to each oyster families under controlled conditions. Experimental infections were performed with infectious environments from Atlantic and Mediterranean origin. This figure was adapted from de Lorgeril et al. (2020) ((https://doi.org/10.1186/s12864-020-6471-).

Biparental oyster families were produced by *in vitro* fertilization from wild genitors sampled in farming and non-farming areas as previously described (de Lorgeril et al., 2018, 2020). Briefly, wild genitors used to produce Atlantic families F9 and F11 were collected at two sites approximately 20 km apart, in Logonna Daoulas (lat.: 48.335263 long.: -4.317922, farming areas) and Dellec (lat.: 48.353970 long.: -4.566123, non-farming areas), respectively (Figure 1A). Wild genitors used to produce Mediteranean families F32 and F44 were collected at two sites approximately 40 km apart, in Vidourle (lat.: 43.553906 long.: 4.095175, non-farming areas) and Thau lagoon (lat.: 43.418736 long.: 3.622620, farming areas), respectively. Genitors coming from aquaculture areas were assumed to be exposed to stronger selection pressure due to mass mortality outbreaks occurring annually at these sites. The last family, F21, was generated from a pair of broodstocks derived from mass selection conducted in the field during four generations in an aquaculture area at the Atlantic coast (La Tremblade, lat 45.781741 long -1.12191) (Degremont et al., 2015).

### Experimental Infections

Our experimental infection protocol consists of a cohabitation between *C. gigas* oysters (“donors”) carrying the disease and “pathogen-free” *C. gigas* oysters (“recipients”) (Petton et al., 2013; de Lorgeril et al., 2018). The “pathogen-free” status of the animals was confirmed by (i) the absence of OsHV-1 DNA detection by qPCR and (ii) a low Vibrio presence (∼10 cfu^–1^ tissue) determined by isolation on selective culture medium (thiosulfate-citrate-bile salts-sucrose agar, TCBS). Oysters were observed to remain free of any abnormal mortality throughout the larval stage until the beginning of the experimental infections. A first experimental infection used donors previously exposed to the infectious environment of Atlantic origin. The donors were “pathogen-free” oysters (mixture of 116-day-old oysters from the 15 families, 17,700 g with a mean individual weight of 1.1 g) were first deployed in a farming area (Logonna Daoulas, lat 48.335263, long - 4.317922) during the infectious period until the first mortalities occurred. Then, donor oysters were transferred back to the laboratory and placed in contact with “pathogen-free” recipient oysters in a controlled environment (Figure 1B). The experiment was conducted using the same biomass (1120 g) of donors in cohabitation in independent tanks, each containing one of the recipient oyster families (1120g with a mean individual weight of 1.1 g) which were previously acclimatized in these structures for 2 weeks. The Atlantic experimental infection began on 17 July 2015 and ended on 31 July 2015. Similarly, a second experimental infection used donors previously exposed to the infectious environment of Mediterranean origin in a farming area (Thau lagoon, lat 43.418736, long 3.622620), except that donors deployed were a mixture of 176-day-old oysters from the 15 families (26,500 g with a mean individual weight of 1.7 g) and that the biomass of donors and the biomass of recipients in each tank was 1760 g each (recipient oysters with a mean individual weight of 1.73 g). The Mediterranean experimental infection began on 21 September 2015 and ended on 6 October 2015. In parallel and for each experimental infection, a control cohabitation experiment was performed under identical conditions but using donors that had not spent time in the farming areas. Mortality was monitored in laboratory tanks. During the experimental infection, 10 oysters in triplicate were randomly sampled without blinding protocols from each tank and at each time point (0, 6, 12, 24, 48, 60, and 72 h). The shell was removed, and pools of 10 oysters were flash frozen in liquid nitrogen.

### DNA and RNA Extractions

Oyster pools (10 individuals per pool) were ground in liquid nitrogen in 50 mL stainless steel bowls with 20 mm diameter grinding balls (Retsch MM400 mill). The obtained powders (stored at −80°C) were then used for extraction of DNA and RNA as previously described in de Lorgeril et al. (2018). Briefly, DNA and RNA were extracted using the NucleoSpin^®^ Tissue Genomic extraction kit (Macherey-Nagel) and the Direct-Zol RNA Miniprep kit (Proteigene), respectively, according to the manufacturer’s protocol. Nucleic acids concentration and purity were checked using a NanoDrop ND-1000 spectrometer (Thermo Scientific), and their integrity was analyzed by capillary electrophoresis on a BioAnalyzer 2100 (Agilent).

### Viral Load Quantification

Quantification of OsHV-1 has been carried out using quantitative PCR (qPCR) and used as a proxy for infection. All amplification reactions have been analyzed using the Roche LightCycler 480 Real-Time thermocycler in three technical replicates (qPHD-Montpellier GenomiX platform, Montpellier University, France). The total qPCR reaction volume was 1.5 μL, consisting of 0.5 μL of DNA (40 ng/μL) and 1 μL of LightCycler 480 SYBR Green I Master mix (Roche) containing 0.5 μM of PCR primer (Eurogenetec SA). Virus-specific primer pairs target the region of the OsHV-1 genome predicted to encode a catalytic subunit of DNA polymerase (ORF100, AY509253): Fw-5′-ATTGATGATGTGGATAATCTGTG-3′ and Rev-5′-GGTAAATACCATTGGTCTTGTTCC-3′ (Webb et al., 2007). A Labcyte Acoustic Automated Liquid Handling Platform (ECHO) has been used for pipetting into the 384-well plate (Roche). A LightCycler^®^ 480 Instrument (Roche) was used for qPCR with the following program: enzyme activation at 95° C for 10 min followed by 40 denaturation cycles (95° C, 10 s), hybridization (60° C, 20 s) and elongation (72° C, 25 s). To check the specificity of the amplification, a subsequent melting temperature curve of the amplicon was performed. For absolute quantification, DP amplification products were cloned into the pCR4-Topo vector and replicated in *Escherichia coli* DH5α (Invitrogen). Plasmids were extracted using the Wizard Plus SV miniprep DNA purification system (Promega) and standard curves of known concentration of plasmid generated according to the following formula [amount of plasmid DNA (ng) × 6.022.10^23^]/[length of DNA template (bp) × 10^9^ × 660]. Absolute quantification of viral DNA copies was then calculated by comparing the reported Cq values to the standard curve constituted of 8 serial dilution points of the circular plasmid (10^9^–10^2^ copies per μL) in three technical replicates (*R*^2^ = 0.993, efficiency = 1.91 and equation: y = -3.5416x + 40.02). Samples were considered positive when superior to 10^2^ copies per μL (corresponding to 30 cycles) and but also when inferior to 10^2^ copies per μL if the melting temperature of amplicon was correct (between 30 and 35 cycles).

### RNA-Seq Analyses

RNA-Seq library construction and sequencing were performed by the Fasteris Company (Switzerland). Directional cDNA libraries were constructed using a TruSeq mRNA Stranded kit (Illumina) and sequenced on a Hiseq in paired-end reads of 2 × 75 bp. Adapters were cleaned using trimmomatic 0.39 (Bolger et al., 2014). The quality of the sequences was evaluated by FastQC v0.11.8 (Andrews, 2010) and reads with a mapping quality above 20 were retained. PCR duplicates from sequencing where remove using picard v2.18.14 (“Picard toolkit,” 2019)^1^ with the MarkDuplicates option. Then reads were aligned to the OsHV-1 μVar A genome (GenBank: KY242785.1) (Burioli et al., 2017) using Bowtie 2 v2.3.4.3 (Langmead and Salzberg, 2012). The abundance of OsHV-1 μVar reads was counted using samtools v1.9 (Li et al., 2009) and normalized by multiplying raw mapped reads on the OsHV-1 μVar A genome by a library normalization factor (calculated as the average library size for all times and controls divided by the library size for the specific time point) (Supplementary Table S1). The abundance of OsHV-1 μVar reads was used as a proxy for viral replication. Data treatments were carried out under a High-performance computing (HPC) cluster using a self-scripted pipeline (code including parameters used are available on https://github.com/IHPE/DivOsHV).

### SNP Calling and Quantification

SNP detection and quantification were performed on families F11 and F32 in both the Atlantic and Mediterranean experimental infections, while families F9 and F44 were only analyzed in the Atlantic experimental infections due to the lower read coverage in the Mediterranean infections. Sequencing reads were mapped to OsHV-1 μVar A genome as described previously. Because the sequencing depths differ between time-points and according to the sensitivities of infected oysters, we first concatenated the three replicates of the three time-points containing the highest viral loads together (48, 60, and 72 h) using samtools merge v1.9 (Li et al., 2009). Then pysamstats v1.1.2^2^ was used to count the nucleotide composition on all genomic positions. SNPs were detected with an *ad hoc* script^3^, using the R language R Core Team, applied to each pysamstats output. Nucleotide changes were called “SNP” if (i) the alternative base frequency was greater than 5%. (ii) the sequencing depth was greater than 180 reads, to insure an average coverage of 60 for the triplicates. (iii) the depth of the alternative base was greater than an intrinsic parameter to the library (mean + standard deviation of all alternative bases) which is used to eliminate all low abundant alternative bases. The outputs were stored in VCF format which allow us to determine the impact of each SNP with SnpEff and SnpSift (Cingolani et al., 2012). Finally, to compare the environments, we combined the data according to the experimental infection origin. SNP counts were summed by family and allelic frequency recalculated using R. All the graphics were made using the ggplot2 package (Wickham, 2016) and Venn diagrams were made with the Venn Diagram package (Chen and Boutros, 2011).

### Statistical Analysis

OsHV-1 colonization was assessed before the first mortalities appear, at 12/24/48/60/72 h post-infection. For the statistical analyzes, the time points were subdivided into two groups, the first one corresponded to the early stage of the infection (12/24 h) that is characterized by an intense increase of the viral load, whereas the second one corresponded to the late stage of the infection (48/60/72 h) when viral load reached a plateau. Mann–Whitney *U*-tests were used (GraphPad Prism 8.4.2) to compare OsHV-1 load and replication rate between families at late phase of the infection (48/60/72 h) (Supplementary Table S2). Mann–Whitney *U*-tests were also used to compare OsHV-1 viral load within each family between early and late phase (Supplementary Table S2).

## Results

### Susceptibility of Oyster Families to POMS Is Associated With Viral Load and Varies According to Disease Origin

In this study, we used five biparental families of juvenile oysters with contrasting phenotypes toward POMS. These families were produced from genitors that had experienced different selective filters (see “Materials and Methods” section). The 5 families were subjected to an experimental infection mimicking disease transmission in nature. For that we performed cohabitation experiments with oysters previously immersed in Atlantic and Mediterranean oyster farms during POMS events (see Figure 1B and “Materials and Methods” section).

**FIGURE 2:**
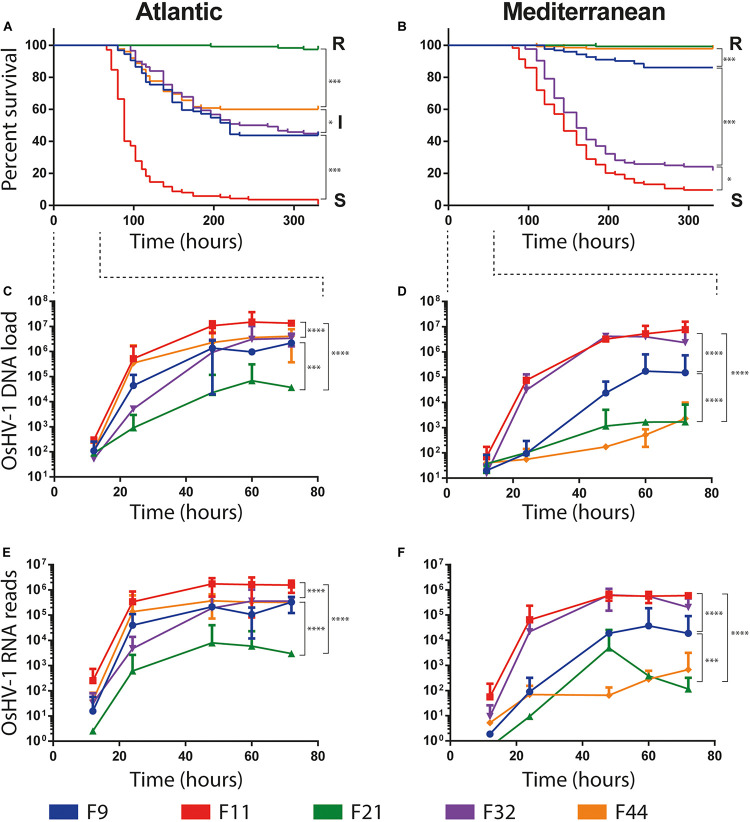
Survival curves and OsHV-1 colonization of five biparental oyster families submitted to experimental infections using donors oysters deployed in Atlantic and Mediterranean farming area during infectious period. **(A,B)** Represent Kaplan-Meier survival curves of the 5 families of recipient oysters during the Atlantic and Mediterranean natural experimental infection, respectively. Resistant, intermediate and susceptible oyster families are indicated by R, I and S, respectively. The stars indicate *P*-values according to Mantel-Cox Log-rank test (^∗^*P* ≤ 0.05, ^∗∗∗^*P* ≤ 0.001). OsHV-1 colonization was assessed at the early time point of experimental infection at 12/24/48/60/72 h. **(C,D)** OsHV-1 load was quantified by qPCR and expressed as viral gene copy number per ng of oyster DNA. **(E,F)** Viral replication was estimated by the total number of RNA-Seq reads mapped on the OsHV-1 μVar A genome (KY242785). The stars indicate *P*-value according to Mann-Whitney multiple comparisons *U*-tests of viral load **(C,D)** and viral replication rate **(E,F)** between families at late phase of infection (48/60/72 h) (^∗∗∗^*P* ≤ 0.001 ^*⁣*⁣**^P ≤ 0.0001).

A high variability in survival rates was observed between families within each experimental infection, ranging from 1 to 98% and 10 to 99.7% in Atlantic and Mediterranean experimental infection, respectively (Figures 2A,B). Two families, F11 and F21, had the same phenotypes, susceptible (S, percentage of survival < 25%) and resistant (R, percentage of survival > 75%), respectively, whatever the experimental infection they faced (Atlantic or Mediterranean). Conversely, the phenotype of the three other families changed with experimental infections. Oyster families F9, F32, and F44, which had an intermediate phenotype (I, 25% ≤ percentage of survival ≥ 75%) in the Atlantic experimental infection (Atl), were found susceptible (F32) or resistant (F9 and F44) in the Mediterranean experimental infection (Med). These phenotypic changes cannot be attributed to a decrease in the severity of POMS in Med since (i) the F11 family had almost the same survival rate in both experimental infections and (ii) the F32 family has a lower survival rate in Med than in Atl.

The load and transcriptional activity of OsHV-1 μVar were monitored within the first 72 h post-infection in the five oyster families and two experimental infections (Atl and Med) using qPCR and RNA-Seq, respectively (Figures 2C–F). In all conditions we observed that the viral loads significantly increased between the early (12/24 h) and the late (48/60/72 h) phase of infection (Figures 2C,D; Mann–Whitney *U*-test, *P* < 0.01 in Atl and *P* < 0.001 in Med; details in Supplementary Table S2). We also detected viral transcripts in both experimental infections for all oyster families (Figures 2E,F). These results show that OsHV-1 was able to infect all oyster families, indicating all were tolerant to infection. However, the amount of viral nucleic acid varied with disease expression: susceptible families accumulated 10^2^–10^3^ times more viral transcripts than resistant ones (Supplementary Table S3). Taken together, these results show that all families were infected with the OsHV-1 μVar virus but the most resistant ones controlled viral replication.

Noteworthy, oyster families with an intermediate phenotype in Atl showed an R or S phenotype in Med. As the major difference between these two experiments is the geographic origin of the infection, it suggests that the POMS occurring on the Atlantic coast is different from that of the Mediterranean coast. This difference in survival phenotypes is also visible at the late phase of infection (48/60/72 h) both in terms of viral load and viral replication rate. Indeed, in Atl, viral load and viral replication rate are not significantly different for the three intermediate phenotype families (F9, F32, and F44, Mann–Whitney *U*-test, *P* > 0.05) (Figures 2C–E and Supplementary Table S2). Conversely, in Med, the viral load and replication rate are significantly different between the three families (Mann–Whitney *U*-test, *P* < 0.001) (Figures 2D–F and Supplementary Table S2). These observations led us to hypothesize that oysters encountered different OsHV-1 μVar populations in the two environments.

### Two Distinct Viral Populations in Atlantic and Mediterranean Infections Revealed by SNPs

**FIGURE 3:**
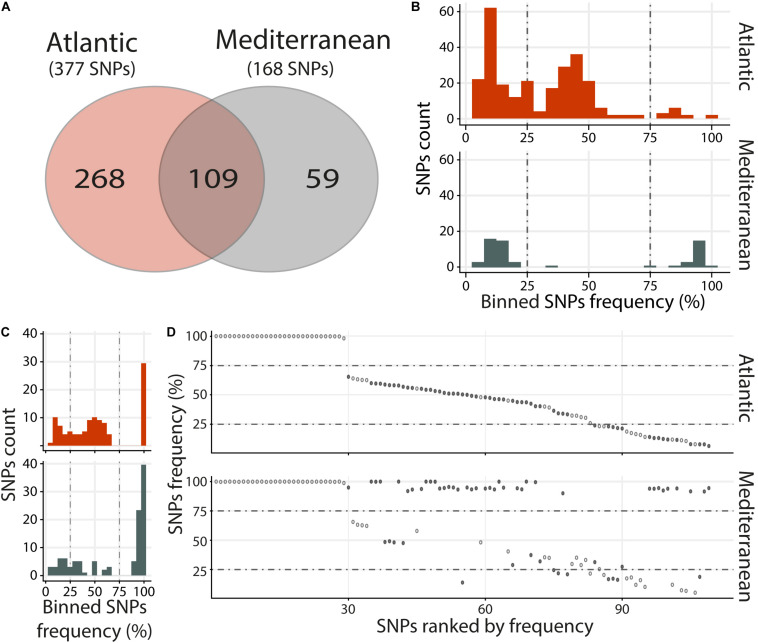
Distinct heterogeneous viral populations within Atlantic and Mediterranean experimental infections. **(A)** Venn diagram representation of the number of specific and common SNPs within each experimental infection. The frequency distribution of SNPs for specific **(B)** and common **(C)** SNPs between Atlantic and Mediterranean experimental infections is illustrated in histograms. SNPs are binned at 5% intervals along the *x*-axis, and the number of SNPs in each bin is indicated on the *y*-axis. **(D)** Common SNPs are ordered according to decreasing variant frequency in Atlantic experimental infection and plotted for the same position in Mediterranean experimental infection. Black and white dots correspond to SNPs with different or similar variant frequencies (difference < 5%), respectively.

Viral populations present in the Atlantic and Mediterranean environments, were analyzed by merging RNA-Seq data according to environments. This included the RNA-Seq reads mapping against OsHV-1 μVar A genome in the 3 replicates and the 3 time points containing the highest viral loads (48, 60, and 72 h), both in intermediate and susceptible families (resistant families were not included in the analyses due to the low number of viral reads). Then, single nucleotide polymorphisms (SNPs) were analyzed. We identified 436 variable positions along OsHV-1 μVar A genome: 268 and 59 were specific to Atl and Med, respectively, and 109 were common to both experimental infections as compared to OsHV-1 μVar A (Figure 3A and Supplementary Tables S4, S5).

Variants were binned (5% intervals) according to their frequency (Figures 3B,C). Variants specific to Atl may be subdivided into three groups, minor (47%; frequency < 25%) intermediate (48.1%; 25% ≤ frequency ≤ 75%) and major variants (4.9%; frequency > 75%) (Figure 3B, upper panel). Variants specific to Med segregated in 2 main clusters, minor (62.7%) and major variants (35.6%) (Figure 3B, lower panel). This difference in distribution of variant frequency from Atl and Med was also observed for variants common to both experimental infections (Figure 3C). Indeed, the percentages of minor and intermediate variants were higher in Atl than in Med (minor: 23.9% and 17.4%, respectively, intermediate: 49.5 and 21.1%, respectively). Conversely, the percentage of major variants was lower in Alt than in Med (26.6 and 60.5%, respectively).

Finally, we compared the variant frequency between Atl and Med for each of the common SNPs on the 109 positions spread all over the OsHV-1 μVar genome (Figure 3D and Supplementary Table S6). On these 109 common SNPs, 49.5% (54 SNPs) had the same variant frequency (difference in variant frequency < 5%) and 50.5% (55 SNPs) had different variant frequency (ranging from 5.1 to 88.2%; median of 42.9%).

Altogether, these results show that the viral populations collected in the two environments (Atlantic and Mediterranean) are different from each other but also different from the OsHV-1 μVar A genome used as reference. Interestingly, the viral population from Med have a reduced genetic diversity compared to the one from Atl. Indeed, whereas viral population from Med had less SNPs, 52.3% of them correspond to major variants with 9.5% of fixation (variant frequency 100%) whereas in Atl, there were only 11.1% of major variants with 1.3% of fixation (Supplementary Tables S4, S5).

### Atlantic and Mediterranean OsHV-1 μVar Viral Populations Bear Different Membranes Proteins

**FIGURE 4:**
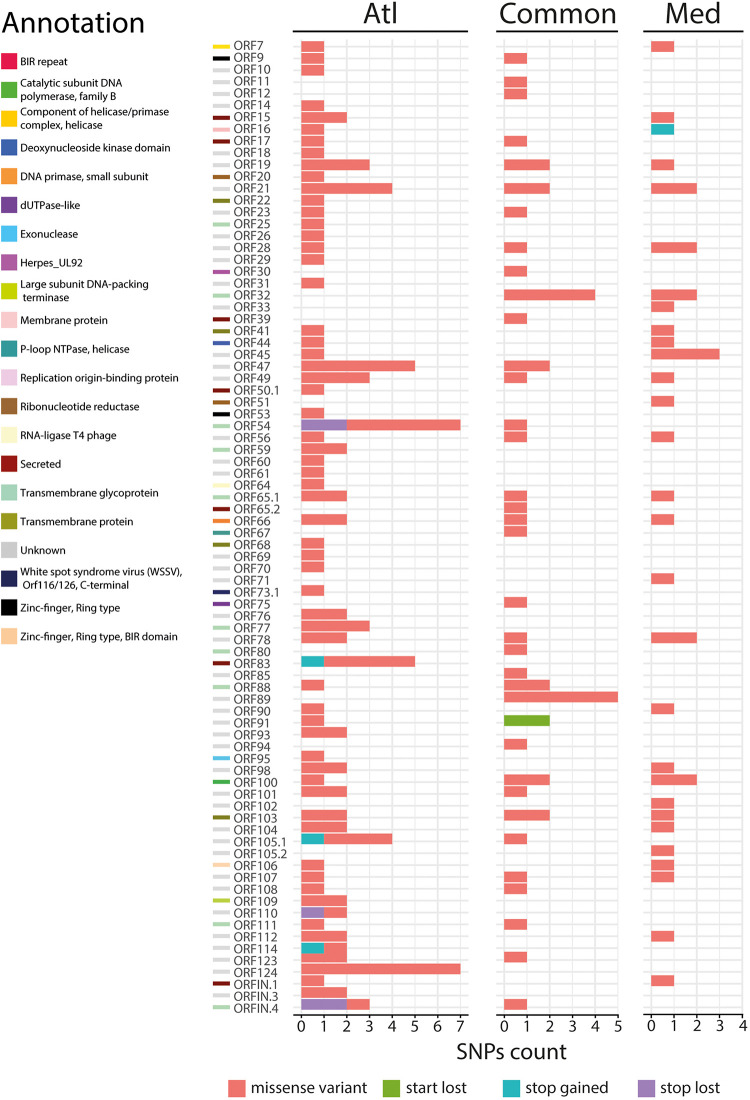
Impact of the 200 nonsynonymous SNPs on the sequence of OsHV-1 μVar ORFs. Stacked barplot indicates the number of nonsynonymous SNPs (*x*-axis) located in each OsHV-1 ORF (*y*-axis). Each color indicates a specific mutation effect as indicated at the bottom of the figure. Each line is related to one of the expressed OsHV-1 ORFs. ORF colors (left side) correspond to a functional annotation of the proteins that they encode.

We further examined the distribution of all variants that occurred in ORFs and we found that 299 SNPs (out of 436) were located in ORFs. Among them, 99 correspond to synonymous SNPs and 200 to nonsynonymous SNPs (nsSNPs) that could be divided into 191 missense variants, 5 stop lost, 4 stop gained, and 2 start lost (Figure 4). The comparison of specific SNPs for each experimental infection revealed that for the Atlantic viral population, 76 ORFs (59.4%) carry at least one nsSNPs compared to only 53 ORFs (41.4%) for the viral populations from the Mediterranean.

We then focused on the nature of the proteins encoded by the ORFs carrying nsSNPs. The most represented ORF group, carrying 55.4% of the nsSNPs, encodes proteins of unknown functions (44 ORFs). The second most represented ORF group encodes membrane-related proteins (membrane and transmembrane proteins, glycosylated or not) and 15 out of 19 ORFs encoding this type of proteins carry 23.3% of the nsSNPs (Table 1). Some ORFs carry numerous nsSNPs (ORF32, ORF54 and ORF103) and were differentially mutated in the two experimental infections. For instance, ORF54 which is predicted to encode a transmembrane glycoprotein of 807 aa carries 8 nsSNPs, 7 of which are specific to Atl. Among these 7 snSNPs, 2 induce a stop_lost, which increases the protein size by 39 aa, and 5 correspond to missense-variants. These mutations in ORFs could impact protein functions.

**TABLE 1:** ORFs encoding membrane related proteins carrying nsSNPs.

**ORF (GenBank accession #)**	**ORF size (in bp)**	**Protein size (in aa)**	**Protein putative function**	**SNPs position**	**Type of variation**	**Allel frequency (in %)**		**Experimental infection**	**Impact on protein**
						**Atl.**	**Med.**		
**ORF16 (ASK05544.1)**	**231**	**76**	**Membrane protein**	21,892	missense_variant	43.00	0.00	Atlantic	His22Gln
				21,917	**stop_gained**	0.00	**76.09**	**Mediterranean**	**deletion 45 aa**
ORF22 (ASK05550.1)	4899	1632	Transmembrane protein, 1 helix	29,343	missense_variant	21.46	0.00	Atlantic	Val64Leu
ORF25 (ASK05553.1)	666	221	Transmembrane glycoprotein, 1 helix	39,333		41.52	0.00	Atlantic	Arg216Lys
ORF32	1659	552	Transmembrane glycoprotein, 1 helix	47,348	missense_variant	51.63	94.55	Common	Ser10Phe
				48,888		14.04	10.18	Common	Leu523Phe
				48,884		10.54	6.78	Common	Trp522Leu
				48,885		11.18	7.28	Common	Trp522Phe
				48,220		0.00	95.11	Mediterranean	Asp301Asn
				48,283		0.00	11.20	Mediterranean	Asp322Asn
ORF41 (ASK05566.1)	2922	973	Transmembrane protein, 1 helix	56,584	missense_variant	48.05	0.00	Atlantic	Asp866Asn
				54,968		0.00	19.09	Mediterranean	Val327Asp
ORF54 (ASK05581.1)	2424	807	Transmembrane glycoprotein, 1 helix	80,895	missense_variant	100.00	99.75	Common	Ser202Gly
				82,296		7.61	0.00	Atlantic	Asp669Asn
				80,297		45.50	0.00	Atlantic	Asn2Lys
				80,299		42.72	0.00	Atlantic	Thr3Lys
				80,301		15.96	0.00	Atlantic	Val4Ile
				80,302		11.41	0.00	Atlantic	Val4Asp
				82,714	stop_lost	25.36	0.00	Atlantic	addition 39 aa
				82,715		26.20	0.00	Atlantic	addition 39 aa
ORF59 (ASK05586.1)	3243	1080	Transmembrane glycoprotein, 1 helix	87,730	missense_variant	8.58	0.00	Atlantic	Asp332Asn
				87,319		5.97	0.00	Atlantic	Glu195Lys
ORF65.1	1263	420	Transmembrane glycoprotein, 1 helix	95,619	missense_variant	59.47	100.00	Common	Thr201Arg
				95,887		8.90	0.00	Atlantic	Glu112Lys
				96,057		9.74	0.00	Atlantic	Asp55Gly
				95,820		0.00	93.58	Mediterranean	Ser134Leu
ORF68 (ASK05592.1)	2082	693	Transmembrane protein, 1 helix	103,523	missense_variant	41.37	0.00	Atlantic	Asp394Tyr
ORF77 (ASK05600.1)	3795	1264	Transmembrane glycoprotein, 2 helix	115,467	missense_variant	40.22	0.00	Atlantic	Arg3Gln
				117,243		7.41	0.00	Atlantic	Gln595Arg
				115,875		7.46	0.00	Atlantic	Thr139Met
ORF80 (ASK05603.1)	351	116	Transmembrane glycoprotein, 1 helix	123,511	missense_variant	99.93	99.96	Common	Tyr77Phe
ORF88 (ASK05611.1)	2247	748	Transmembrane glycoprotein, 1 helix	130,979	missense_variant	99.95	99.90	Common	Val381Gly
				130,997		99.92	99.86	Common	Thr387Met
				131,902		7.63	0.00	Atlantic	Asp689Asn
ORF103 (ASK05626.1)	1272	423	Transmembrane protein, 4 helix	153,070	missense_variant	40.57	0.00	Atlantic	Glu43Lys
				153,223		47.84	94.06	Common	Gly94Arg
				153,224		48.52	94.31	Common	Gly94Glu
				153,848		22.79	0.00	Atlantic	Ser302Leu
				153,151		0.00	13.53	Mediterranean	Glu70Lys
ORF111 (ASK05633.1)	870	289	Transmembrane glycoprotein, 5 helix	168,846	missense_variant	8.04	0.00	Atlantic	Ser51Leu
				168,853		43.81	94.32	Common	Glu49Lys
**ORF114 (ASK05636.1)**	**969**	**321**	**Transmembrane glycoprotein, 1 helix**	171,530	**stop_gained**	**84.63**	0.00	**Atlantic**	**Deletion 307 aa**
				171,532	p.Ile16Lys	89.40	0.00	Atlantic	Ile16Lys
ORFIN.4 (ASK05572.1)	981	326	Transmembrane glycoprotein, 1 helix	61,244	missense_variant	99.96	99.93	Common	Phe166Ile
				61,725	missense_variant	9.08	0.00	Atlantic	Ser326Leu
				61,728	stop_lost	11.40	0.00	Atlantic	Addition 3 aa
				61,729		12.40	0.00	Atlantic	Addition 3 aa

*The ORFs in bold correspond to variants of the stop_gained type.*

More interestingly, two ORFs carry stop_gained mutations at high frequency. The first one, found specifically in Med at a frequency of 76.1%, truncates the ORF16, predicted to encode a transmembrane protein, by replacing the 31st amino acid by a stop codon removing 58.4% of the protein. The second one, found specifically in Atl at a frequency of 84.6%, truncates the ORF114 by replacing the 15th amino acid by a stop codon removing 95.3% of the protein. Amino acid sequence analysis predict that ORF114 encodes a transmembrane glycoprotein with a transmembrane helix (aa 13–35) and a glycosylated extracellular domain (aa 36–322), both of which are truncated by the mutation (Supplementary Figure S1). Interestingly, size variations of these ORFs have already been reported for the different OsHV-1 genomes available. The most striking size variations are observed for the protein encoded by the ORF114 with 494 aa in OsHV-1 (Davison et al., 2005) 322 in OsHV-1 μVar A (Burioli et al., 2017) and 282 in OsHV-1-PT (Abbadi et al., 2018). Surprisingly, whereas the three proteins are almost 100% identical on their C-terminal part (281 aa), solely the protein encoded by OsHV-1 μVar A contains a transmembrane helix (Supplementary Figure S2; Burioli et al., 2017).

### Oyster Families Are Infected by Distinct Viral Populations in a Same Environment

In order to determine if the different oyster families are infected or not by the same viral populations within each experimental infection, we compared, independently in experimental infection, the number and frequency of SNPs between oyster families with intermediates and/or susceptible phenotypes.

**FIGURE 5:**
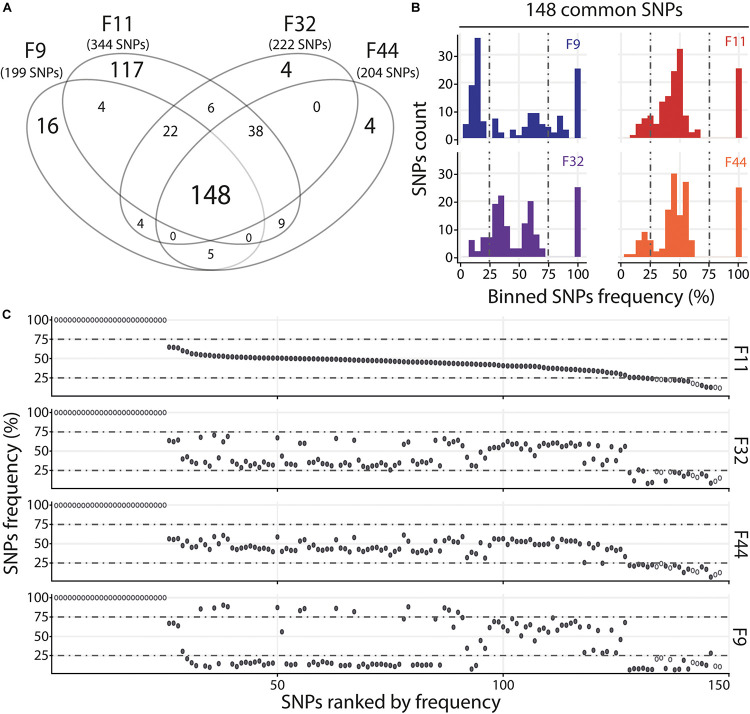
Distinct heterogeneous viral populations infect oyster families during Atlantic experimental infection. **(A)** Venn diagram representation of the number of specific and common SNPs in viral populations infecting each susceptible or intermediate oyster families (F9/11/32/44). **(B)** The frequency distribution of common SNPs across the 4 infected families is illustrated by a histogram. SNPs are binned at 5% intervals along the *x*-axis, and the number of SNPs in each bin is indicated on the *y*-axis. **(C)** Common SNPs are ordered according to decreasing variant frequency in F11 family and plotted for the same position in F32, F44, and F9 families. Black and white dots correspond to SNPs with different or similar variant frequencies (difference < 5%), respectively.

For the Atl, we compared the four oyster families with susceptible (F11) and intermediate (F9, F32, and F44) phenotypes. We observed that families with intermediate phenotypes contained a similar number of variants whereas the susceptible family F11 contained a larger number of variants (Figure 5A and Supplementary Table S7). This difference could be related to the fact that susceptible families have a higher viral load than the families with intermediate phenotype. The higher number of viral reads obtained in susceptible oysters could have facilitated the identification of viral variants. However, each family with intermediate phenotypes (F9, F32, and F44) contained specific SNPs which were not found in F11, which suggests viral populations differ between oyster families. We also identified 148 SNPs common to the 4 families. The analysis of F11/32/44 revealed that their variant frequency are equally scattered with 12–15% of minor variants, 68–71% of intermediate variants and 17% of major variants (Figure 5B and Supplementary Table S7). For the F9 we observed a majority of minor variant (45%), followed by 30 and 26% of intermediate and major variants, respectively (Figure 5B and Supplementary Table S7). We also compared the variant frequency between the four oyster families for each of the common SNPs on the 148 positions spread all over the OsHV-1 μVar genome (Figure 5C). On these 148 common SNPs, only 12.2% (32) have the same variant frequency in the four families (difference in variant frequency < 5%). A two by two comparison of the four families revealed that the percentage of SNPs having the same variant frequency ranked from 29% (43 SNPs for F9/F11) to 46% (68 SNPs for F32/F44) (Supplementary Table S8). These results show that the four families of oyster do not contain the same viral populations.

**FIGURE 6:**
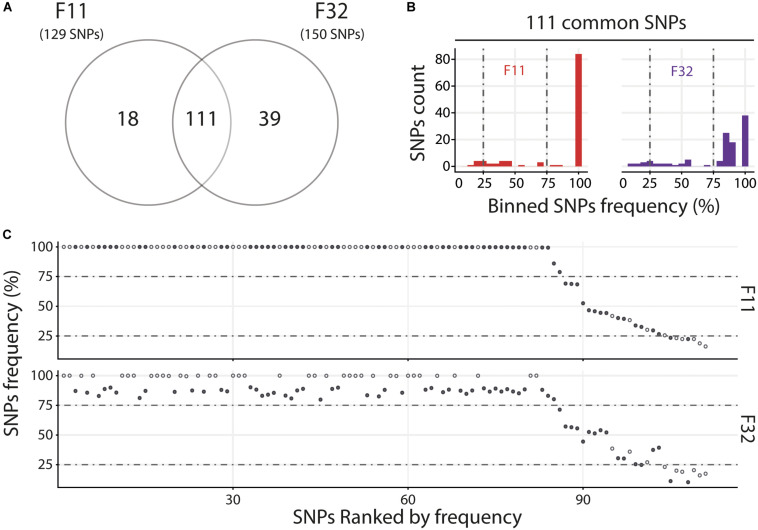
Distinct heterogeneous viral populations infect oyster families during Mediterranean experimental infection. **(A)** Venn diagram representation of the number of specific and common SNPs in viral populations infecting each susceptible oyster families (F11/32). **(B)** The frequency distribution of common SNPs across the 2 infected families is illustrated by a histogram. SNPs are binned at 5% intervals along the *x*-axis, and the number of SNPs in each bin is indicated on the *y*-axis. **(C)** Common SNPs are ordered according to decreasing variant frequency in F11 family and plotted for the same position in F32 family. Black and white dots correspond to SNPs with different or similar variant frequencies (difference < 5%), respectively.

For the Med, we compared the two oyster families with susceptible phenotypes (F11 and F32) and observed that they contain a similar number of variants with 111 common and 18 and 39 specific to F11 and F32, respectively (Figure 6A). Variant frequency analysis of the 111 common SNPs revealed that they are equally distributed in the three groups of variants (Figure 6B and Supplementary Table S9). Indeed, there was 6.3 and 8.1% of minor variants in F11 and F32, respectively, 16.2 and 15.3% of intermediate variants in F11 and F32, respectively, and 77.5 and 76.6% of major variants in F11 and F32, respectively. We also compared the variant frequency between the two oyster families for each of the common SNPs on the 111 positions spread all over the OsHV-1 μVar genome (Figure 6C and Supplementary Table S10). On these 111 common SNPs, 47 have the same variant frequency (difference in variant frequency < 5%). For the other SNPs, it is interesting to note that the variant frequency of 91% of them (58 out of 64) is higher in F11 than in F32. In addition, the number of fixed SNPs (variant frequency 100%) is almost twice higher in F11 (41) than in F32 (24). These results also showed that F11 and F32 do not contain the same viral populations in this environment.

Altogether, these results show that in as same environment, each oyster family is infected by distinct viral populations.

## Discussion

In the present study, we analyzed the OsHV-1 diversity in oysters with different genetic backgrounds exposed to two different environments (Atlantic and Mediterranean oyster farming areas during an episode of POMS). The two infection experiments led to contrasting survival phenotypes between oyster families within and between environments. As OsHV-1 infection is the first and necessary step to induce POMS (de Lorgeril et al., 2018) we focused our study on the diversity of OsHV-1 populations infecting oysters.

Viral sequences found in oyster tissues differed between environments with an overall number of 436 SNP variants identified. Sequences differed from the OsHV-1 μVar A genome (originating from the English Channel, Normandy coast, France) used here as reference (Burioli et al., 2017). Moreover, viral populations from the Atlantic and the Mediterranean differed (i) by 268 SNPs specific to the Atlantic environment, 59 specific to the Mediterranean environment, and also (ii) by variant frequencies within the 109 SNPs common to both environments. SNP abundance was twice higher in the Atlantic environment with a fixation rate seven times lower compared to the Mediterranean environment, which suggests that the viral populations infecting oysters in the Atlantic are more diversified than in the Mediterranean Sea. One possible environmental reason for this difference is that in the Atlantic, oyster farms are located in the Brest harbor, which is colonized by a huge population of wild oysters cohabitating with farmed oysters. In contrast in the Mediterranean Thau lagoon, farmed oysters represent the majority of the oyster population (Lejart, 2009; Pernet et al., 2012). The wild oyster populations of the Atlantic are likely to be reservoirs of viruses with a higher genetic diversity than the cultured populations of the Thau lagoon, as indicated by previous studies showing a lower viral diversity in hatchery−reared oysters relative to wild individuals (Vendrami et al., 2019). In full support of these observations, a higher host genetic diversity is expected to generate a higher viral diversity since viruses are obligatory cellular parasites, which implies that the virus and the host partners reciprocally affect each other’s evolution (Kajan et al., 2019). Regardless of the origin of this diversity, our analysis revealed that the OsHV-1 μVar diversity along French coasts is higher than previously described (Burioli et al., 2018). This result is consistent with the high diversity also observed for this virus along the Italian Adriatic Sea coast (Burioli et al., 2016).

To further analyze the different viral populations infecting the different oyster families, we compared the OsHV-1 diversity between each oyster genetic background in a same environment (Atl or Med). We observed that (i) each oyster family had its own subset of specific variants and (ii) the majority of common variants displayed distinct frequencies between families. Therefore different families (with intermediates and/or susceptible phenotypes) are infected by distinct viral populations. This suggests that each biparental oyster family, with its own genetic background, is compatible with a subset of the viral variants. In other words, each oyster genotype could constitute a transmission bottleneck that may retain a part of the viral diversity present in the viral population. Although the role of genetic bottlenecks in virus transmission is well documented, their impact on viral genetic diversity and host-virus coevolution are difficult to predict (McCrone and Lauring, 2018). Indeed, it depends on numerous parameters like the virus type, the size of the initial viral population, the size of the bottleneck, as well as the relative impact of within and between host processes (Zwart and Elena, 2015). Genome-wide analyses recently highlighted the role played by inter- and intra-host herpesvirus genomic diversity in colonization of the host (new organ or host shift) and disease manifestations (Loncoman et al., 2017; Renner and Szpara, 2018; Akhtar et al., 2019). Defining the oyster and OsHV-1 factors that determine the transmission bottleneck would be an important step to understand the different levels of infectivity observed between the different oyster families. To reach this end, it would be necessary to study, within each individual oyster, the dynamics of OsHV-1 diversity.

In addition to the presence of distinct viral populations in the two environments that differentially infect each oyster genetic backgrounds, we also observed that oyster families may display a different survival phenotype according to the infectious environment they experienced. Three oyster families F9, F32, and F44, which had an intermediate phenotype during the Atlantic experimental infection, were found susceptible or resistant during the Mediterranean experimental infection. This observation suggests that some oyster genotypes are more susceptible to some viral variants than to others. Several parameters such as the environment, viral genomic feature and host genetics might explain these differences in disease outcome.

By investigating SNPs found in viral populations specific to each environment, we uncovered some differences in viral proteins and more specifically in membrane-related proteins. In mammalian *herpesviridae* (i.e., cytomegalovirus) membrane-related proteins and particularly glycoproteins play a critical role in virus entry into host cell or escape of the host immunity and it has been proposed that the intense selection playing on these proteins could be driven by the interaction with the host immune system (Gardner and Tortorella, 2016; Lassalle et al., 2016). Here we found that 15 out of 19 ORFs encoding this type of proteins in the OsHV-1 genome, contain at least one nsSNP and most of these mutations are specific to Atlantic or Mediterranean viral populations. Two of these ORFs (ORF16 and ORF114) are particularly interesting since (i) they correspond to stop-gain mutations inducing large protein deletion and (ii) each of them is specific to a viral population. Previous studies already pointed out mutations in membrane proteins and suggested that they could be involved in the higher virulence of the μVar genotype compared to the reference OsHV-1 genotype (Martenot, 2013; Burioli et al., 2017; Burioli et al., 2018). Interestingly, two other ORFs encoding membrane-related proteins (ORF 25 and ORF 41), which are differentially mutated in Atlantic and Mediterranean viral populations, appeared to be involved in interaction between OsHV-1 and host cells (Martenot et al., 2019). We hypothesize that the mutations in membrane related proteins described in this study could have functional consequences on host-virus interactions and could be linked to the difference in susceptibility observed for the families F9, F32, and F44 when they were confronted to disease originated from Atlantic or Mediterranean environments.

The observed phenotypic heterogeneity could also be related to the genetic background of oysters. Numerous studies already showed the genetic basis for resistance to OsHV-1μVar in oyster (Azema et al., 2015, 2017; Camara et al., 2017; Gutierrez et al., 2018). Recent data further showed that oyster genetic selection for survival to one variant of OsHV-1 may not greatly increase survival to another variant (Divilov et al., 2019) suggesting that the genetic architecture of resistance to OsHV-1 infection in oyster is differentially structured according to viral genotypes involved.

## Conclusion

In conclusion, the results obtained revealed the presence of OsHV-1 populations, distinct from the OsHV-1μVar A genome, are different in the Atlantic and Mediterranean environments but also differentially infect oyster families. These inter-environment and inter-family genetic variations might be indicative of a host-pathogen co-evolutionary process. Although OsHV-1 genotypic variations have been extensively described in the literature, it is the first time to our knowledge, that a potential genotype-genotype interaction is highlighted in oyster-OsHV-1 interactions. The next challenges will be to combine phenotypic measures of viral impact in experimental infections with whole viral genomes sequencing and comparative genomics techniques to make headway toward a better understanding of how specific genetic differences in OsHV-1 and oyster influence the outcome of infection and how this may shape the distribution of genetic variation within and between viral populations. This study highlights the need for future investigations on co-evolutionary dynamics of oyster-OsHV-1 interactions taking into account viral diversity, viral mutation rate through space and time, mechanisms of adaptation in changing environments that will have to be considered in future strategies to mitigate POMS and shape next oyster selective breeding programs.

## Data Availability Statement

The datasets generated for this study can be found in the SRA database BioProject accession number PRJNA423079 with submission ID from SAMN08382938 to SAMN08382973, SAMN13818404 to SAMN13818448 and from SAMN13818479 to SAMN13818553.

## Author Contributions

JL, BP, CM, J-ME, CC, and GM were involved in the study concept and design. BP, YG, CM, JL, J-ME, and GM were involved in the collection of samples and in the experimental work. JD, RG, P-LS, and CC were involved in bioinformatic and statistical analyses. JD, RG, CM, DD-G, GM, and J-ME drafted the manuscript. All authors contributed to critical revisions and approved the final manuscript.

## Conflict of Interest

The authors declare that the research was conducted in the absence of any commercial or financial relationships that could be construed as a potential conflict of interest.

## Footnotes

1 http://broadinstitute.github.io/picard/

2 http://github.com/alimanfoo/pysamstats

3 https://github.com/IHPE/DivOsHV
