# Supplementary material for: Contribution of Viral Genomic Diversity to Oyster Susceptibility in the Pacific Oyster Mortality Syndrome
Source: Front Microbiol. 2020 Jul 10;11:1579. doi: 10.3389/fmicb.2020.01579 (PMC7381293; doi:10.3389/fmicb.2020.01579)
Supplement: Supplementary file 11 [file Presentation_1.PPTX]

## Slide 1
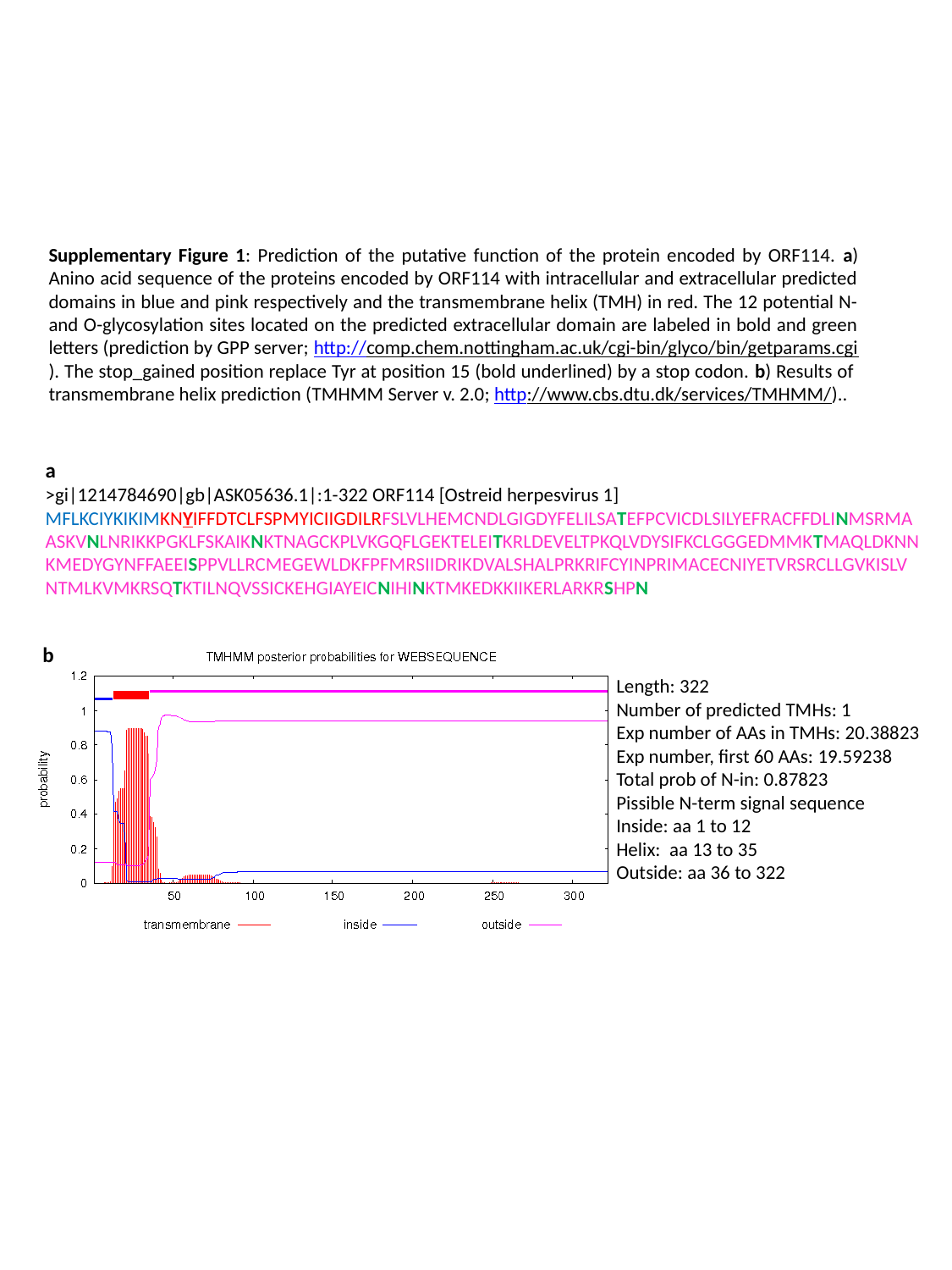

Supplementary Figure 1: Prediction of the putative function of the protein encoded by ORF114. a) Anino acid sequence of the proteins encoded by ORF114 with intracellular and extracellular predicted domains in blue and pink respectively and the transmembrane helix (TMH) in red. The 12 potential N- and O-glycosylation sites located on the predicted extracellular domain are labeled in bold and green letters (prediction by GPP server; http://comp.chem.nottingham.ac.uk/cgi-bin/glyco/bin/getparams.cgi). The stop_gained position replace Tyr at position 15 (bold underlined) by a stop codon. b) Results of transmembrane helix prediction (TMHMM Server v. 2.0; http://www.cbs.dtu.dk/services/TMHMM/)..
a
>gi|1214784690|gb|ASK05636.1|:1-322 ORF114 [Ostreid herpesvirus 1]
MFLKCIYKIKIMKNYIFFDTCLFSPMYICIIGDILRFSLVLHEMCNDLGIGDYFELILSATEFPCVICDLSILYEFRACFFDLINMSRMAASKVNLNRIKKPGKLFSKAIKNKTNAGCKPLVKGQFLGEKTELEITKRLDEVELTPKQLVDYSIFKCLGGGEDMMKTMAQLDKNNKMEDYGYNFFAEEISPPVLLRCMEGEWLDKFPFMRSIIDRIKDVALSHALPRKRIFCYINPRIMACECNIYETVRSRCLLGVKISLVNTMLKVMKRSQTKTILNQVSSICKEHGIAYEICNIHINKTMKEDKKIIKERLARKRSHPN
b
Length: 322
Number of predicted TMHs: 1
Exp number of AAs in TMHs: 20.38823
Exp number, first 60 AAs: 19.59238
Total prob of N-in: 0.87823
Pissible N-term signal sequence
Inside: aa 1 to 12
Helix: aa 13 to 35
Outside: aa 36 to 322
