# Supplementary material for: Contribution of Viral Genomic Diversity to Oyster Susceptibility in the Pacific Oyster Mortality Syndrome
Source: Front Microbiol. 2020 Jul 10;11:1579. doi: 10.3389/fmicb.2020.01579 (PMC7381293; doi:10.3389/fmicb.2020.01579)
Supplement: Supplementary file 12 [file Presentation_2.PPTX]

## Slide 1
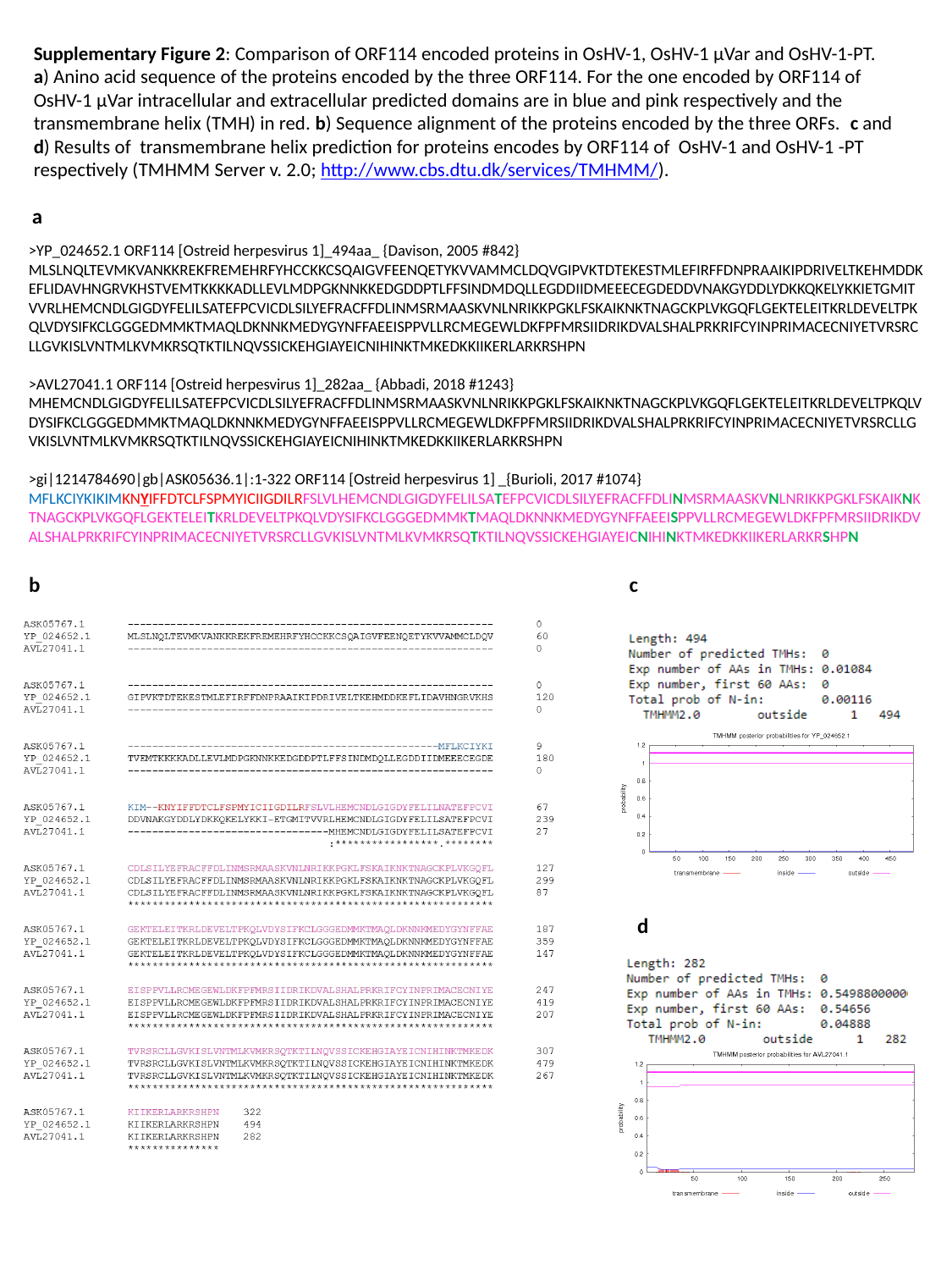

Supplementary Figure 2: Comparison of ORF114 encoded proteins in OsHV-1, OsHV-1 µVar and OsHV-1-PT. a) Anino acid sequence of the proteins encoded by the three ORF114. For the one encoded by ORF114 of OsHV-1 µVar intracellular and extracellular predicted domains are in blue and pink respectively and the transmembrane helix (TMH) in red. b) Sequence alignment of the proteins encoded by the three ORFs. c and d) Results of transmembrane helix prediction for proteins encodes by ORF114 of OsHV-1 and OsHV-1 -PT respectively (TMHMM Server v. 2.0; http://www.cbs.dtu.dk/services/TMHMM/).
a
>YP_024652.1 ORF114 [Ostreid herpesvirus 1]_494aa_ {Davison, 2005 #842}
MLSLNQLTEVMKVANKKREKFREMEHRFYHCCKKCSQAIGVFEENQETYKVVAMMCLDQVGIPVKTDTEKESTMLEFIRFFDNPRAAIKIPDRIVELTKEHMDDKEFLIDAVHNGRVKHSTVEMTKKKKADLLEVLMDPGKNNKKEDGDDPTLFFSINDMDQLLEGDDIIDMEEECEGDEDDVNAKGYDDLYDKKQKELYKKIETGMITVVRLHEMCNDLGIGDYFELILSATEFPCVICDLSILYEFRACFFDLINMSRMAASKVNLNRIKKPGKLFSKAIKNKTNAGCKPLVKGQFLGEKTELEITKRLDEVELTPKQLVDYSIFKCLGGGEDMMKTMAQLDKNNKMEDYGYNFFAEEISPPVLLRCMEGEWLDKFPFMRSIIDRIKDVALSHALPRKRIFCYINPRIMACECNIYETVRSRCLLGVKISLVNTMLKVMKRSQTKTILNQVSSICKEHGIAYEICNIHINKTMKEDKKIIKERLARKRSHPN
>AVL27041.1 ORF114 [Ostreid herpesvirus 1]_282aa_ {Abbadi, 2018 #1243}
MHEMCNDLGIGDYFELILSATEFPCVICDLSILYEFRACFFDLINMSRMAASKVNLNRIKKPGKLFSKAIKNKTNAGCKPLVKGQFLGEKTELEITKRLDEVELTPKQLVDYSIFKCLGGGEDMMKTMAQLDKNNKMEDYGYNFFAEEISPPVLLRCMEGEWLDKFPFMRSIIDRIKDVALSHALPRKRIFCYINPRIMACECNIYETVRSRCLLGVKISLVNTMLKVMKRSQTKTILNQVSSICKEHGIAYEICNIHINKTMKEDKKIIKERLARKRSHPN
>gi|1214784690|gb|ASK05636.1|:1-322 ORF114 [Ostreid herpesvirus 1] _{Burioli, 2017 #1074}
MFLKCIYKIKIMKNYIFFDTCLFSPMYICIIGDILRFSLVLHEMCNDLGIGDYFELILSATEFPCVICDLSILYEFRACFFDLINMSRMAASKVNLNRIKKPGKLFSKAIKNKTNAGCKPLVKGQFLGEKTELEITKRLDEVELTPKQLVDYSIFKCLGGGEDMMKTMAQLDKNNKMEDYGYNFFAEEISPPVLLRCMEGEWLDKFPFMRSIIDRIKDVALSHALPRKRIFCYINPRIMACECNIYETVRSRCLLGVKISLVNTMLKVMKRSQTKTILNQVSSICKEHGIAYEICNIHINKTMKEDKKIIKERLARKRSHPN
c
b
d
